# Digestion Assays in Allergenicity Assessment of Transgenic Proteins

**DOI:** 10.1289/ehp.8803

**Published:** 2006-05-10

**Authors:** Rod A. Herman, Nicholas P. Storer, Yong Gao

**Affiliations:** Dow AgroSciences LLC, Indianapolis, Indiana, USA

**Keywords:** allergy, digestion, risk assessment, simulated gastric fluid, transgenic proteins

## Abstract

The food-allergy risk assessment for transgenic proteins expressed in crops is currently based on a weight-of-evidence approach that holistically considers multiple lines of evidence. This approach recognizes that no single test or property is known to distinguish allergens from nonallergens. The stability of a protein to digestion, as predicted by an *in vitro* simulated gastric fluid assay, currently is used as one element in the risk assessment process. A review of the literature on the use of the simulated gastric fluid assay to predict the allergenic status of proteins suggests that more extensive kinetic studies with well-characterized reference proteins are required before the predictive value of this assay can be adequately judged.

As part of the safety assessment for transgenic crops, the risk for an introduced protein to be a food allergen is considered [Codex Alimentarius Commission 2003; Food and Agriculture Organization of the United Nations/World Health Organization (FAO/WHO) 2001; Goodman et al. 2005; Kimber and Dearman 2002; Mendelsohn et al. 2003; Metcalfe 2005; Metcalfe et al. 1996]. Because no single assay or property can distinguish allergens from nonallergens, a weight-of-evidence approach that holistically considers multiple lines of evidence is used to estimate the risk of allergenicity (Codex Alimentarius Commission 2003; FAO/WHO 2001; Goodman et al. 2005; Mendelsohn et al. 2003; Metcalfe 2005; Metcalfe et al. 1996). The allergenic nature of the organism from which the protein was originally isolated is a primary consideration. If the original source is known to cause allergy, then sera from patients allergic to the source organism are tested for reactivity to the purified transgenic protein. A second major consideration is whether the transgenic protein shares significant structural similarities with known allergens. High structural similarity with a known allergen may indicate shared epitopes for IgE antibody binding and a potential for cross-reactivity and elicitation of allergy (Aalberse 2000; Ferreira et al. 2004; Goodman et al. 2005; Jenkins et al. 2005; Metcalfe 2005; Metcalfe et al. 1996; Stadler and Stadler 2003). Three additional factors relating to exposure level are also often considered: prevalence of the transgenic protein in food, stability of the protein to food processing (Takagi et al. 2003), and digestive stability (Bannon et al. 2002, 2003; Goodman et al. 2005; Kimber and Dearman 2002; Mendelsohn et al. 2003; Metcalfe 2005; Metcalfe et al. 1996). To be useful as indicators of allergenic potential within a weight-of-evidence approach, the relevance of each factor must be understood, and the methods for measuring them must be reproducible and robust. Here we discuss the simulated gastric fluid (SGF) assay and its usefulness in estimating the allergenic risk of dietary proteins.

## Gastric Digestion Assay

Astwood et al. (1996) published a study that suggested a link between the stability of a protein in SGF and its status as a food allergen. SGF is a defined set of reagents held under specific conditions (0.32% pepsin, pH 1.2, 37°C) and was developed to represent human gastric conditions in the stomach (U.S. Pharmacopeia 2000). Although a number of subsequent studies have indicated a much weaker link between stability in SGF and allergenicity (e.g., Diaz-Perales et al. 2003; Fu et al. 2002; Herman et al. 2004; Lee et al. 2005; Murtagh et al. 2002; Vieths et al. 1999; Yagami et al. 2000), the resistance of a transgenic protein to pepsin digestion under acidic conditions is still generally accepted as one factor to be considered in a weight-of-evidence approach for assessing the allergenic risk for transgenic proteins (Bannon et al. 2002, 2003; Codex Alimentarius Commission 2003; FAO/WHO 2001; Goodman et al. 2005; Mendelsohn 2003; Metcalfe 2005). Although SGF assays may not actually mimic *in vivo* digestion, the stability of a protein in SGF is believed to be related to resistance to proteolytic processes that are encountered within the digestive system and/or the intracellular environment (Bannon et al. 2002, 2003; Dearman et al. 2002; Goodman et al. 2005; Huby et al. 2000; Mendelsohn et al. 2003; Metcalfe 2005). Logically, some level of *in vivo* stability must be required for a protein (or a digestion fragment) to interact with the immune system and induce allergy.

## Assay Reproducibility

Because of the inconsistent link between SGF stability and allergenicity that has been seen among different studies, the variation in SGF assay procedures among investigators has been scrutinized as a possible cause for the conflicting conclusions (Bannon et al. 2002, 2003; Metcalfe 2005; Thomas et al. 2004). Differences in pepsin concentration, pH, protein–substrate concentration, and analytical (detection) procedures (SDS–PAGE gel types, loading quantity, protein staining methods, antibody sensitivity for Western blots, etc.) have been considered major confounding factors. Because of this variability among laboratories, there has been a general call for the establishment of a standardized SGF assay procedure (e.g., Bannon et al. 2002, 2003; Metcalfe 2005; Thomas et al. 2004). In response, the International Life Sciences Institute conducted a ring study using identical procedures and reagents to evaluate the reproducibility of a standardized assay when conducted in different laboratories. This study determined that when this specific enzymatic assay was conducted by different researchers with aliquots of the same reagents under similar test conditions, a panel of scientists could identify a similar time for protein bands to become undetectable on SDS–PAGE gels (Thomas et al. 2004). Results validated the reproducibility of this specific enzymatic assay and the technique for detecting the substrate protein.

## Assay Validation

A fully validated assay not only must be reproducible but also must be robust and relevant (Gerberick et al. 2002; Green 1996; National Institute of Environmental Health Sciences 1997; Organisation for Economic Co-operation and Development 2005). A valid SGF assay must be largely insensitive to factors that are likely to vary among laboratories. At a minimum, different preparations of pepsin should yield similar results, and different scientists should be able to interpret results in a similar manner. Furthermore, the use of different analytical techniques for tracking protein digestion should lead to comparable interpretations of stability.

## Kinetic Data Analysis

A great deal of literature exists on the conduct and interpretation of enzyme assays (Anson 1938; Duggleby 2001; Jaswal et al. 2002; Michaelis and Menten 1913; Noda et al. 1994; Park and Marqusee 2004; Rawn 1989; Schnell and Maini 2000; Tzafriri 2003) and biodegradation studies (Alexander and Scow 1989; Atkins 1986; Boesten et al. 2005; Herman and Scherer 2003), including pepsin-mediated digestion (Baderschneider et al. 2002; Boushaba et al. 2003; Bull and Currie 1949; Hollands and Fruton 1968; Swoboda et al. 2001; Tagliazucchi et al. 2005; Tritsch and Sachatello 1971). Thus, we incorporated kinetic concepts into SGF studies conducted by our laboratory (Herman et al. 2003, 2004, 2005). Rather than using a single time point when a protein band was no longer visible on an SDS–PAGE gel or Western blot (time to disappearance based on the human eye), protein bands on SDS–PAGE gels were quantified by densitometry (Bindslev-Jensen et al. 2003; Brussock and Currier 1990; Cantu and Nelson 1994; Syrovy and Hodny 1991) over a digestion time course, and the pattern of protein degradation was modeled using a negative exponential equation (pseudo-first-order decline). Studies on the pepsinolysis of proteins and peptides have often supported a pseudo-first-order pattern of digestion (e.g., Baderschneider et al. 2002; Belorgey et al. 1996; Garrett et al. 2004; Irvine et al. 1983; Matthyssens et al. 1972; Sachdev and Fruton 1975; Terada at al. 1974).

Exponential decline is one of the most common patterns seen for biodegradation and allows a single descriptor, half-life, to be used to characterize the pattern of stability (Alexander and Scow 1989; Boesten et al. 2005; Herman and Scherer 2003; Palasanthiran et al. 1994; Ramanathan 1997; Rawn 1989; Spiess et al. 1996). This measure of digestive stability is independent of many of the factors previously identified as variable among laboratories (e.g., type of protein stain, gel type, loading quantity) because it measures relative amounts of surviving protein rather than a combination of the absolute amount of protein remaining and the specific detection level for that protein (binding affinity of dye for the specific protein and gel loading amounts; Herman et al. 2003; Tal et al. 1980). Thus, a kinetic approach to analysis of degradation results uses multiple data points and relative protein decline to overcome some of the shortcomings associated with observing the first time point at which a protein is no longer visible to the human eye (time to disappearance). It is a standard practice to characterize the specific activity of pepsin using kinetic experiments (Anson 1938; Astwood et al. 1996; Thomas et al. 2004), and we extended this general concept to evaluations of proteins being investigated for stability in SGF (Herman et al. 2003, 2004, 2005).

## Digestion Fragments and Protein Fractions

Interpretation of SGF results is sometimes complicated by the appearance of digestion fragments (large peptides). These fragments may be more persistent than the parent protein substrate. Because no minimum exposure threshold has been established for food allergens (Bindslev-Jensen et al. 2002), and because it is believed that more stable proteins represent a greater allergenic risk, researchers have focused on the most persistent protein fragment when assessing allergenic risk (e.g., Astwood et al. 1996; Fu et al. 2002; Herman et al. 2004; Metcalfe et al. 1996). Similarly, when multiple kinetic phases of digestion were seen for a single protein substrate, our laboratory used the slower, terminal, exponential phase of digestion to evaluate stability in SGF (Herman et al. 2004, 2005). This latter approach does not differ conceptually from the time-to-disappearance approach for evaluating stability in SGF that focuses qualitatively on the terminal data point (where protein bands are no longer visible). Thus, the most persistent digestion fragment or protein fraction has consistently been used to evaluate allergenic risk.

## Assay Robustness

**Figure 1:**
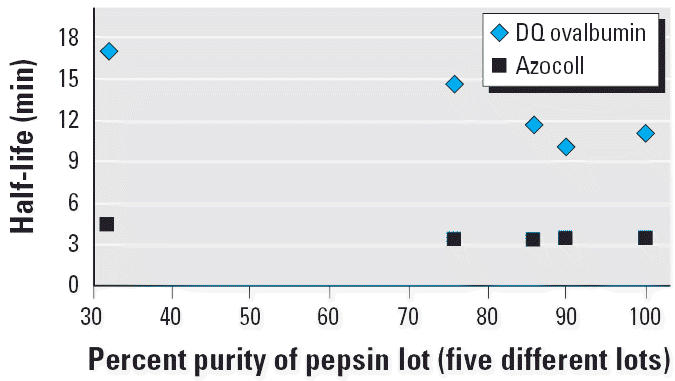
Stability of different lots of pepsin: half-lives for the reporter substrates DQ ovalbumin (50 μg/mL) and azocoll (500 μg/mL) when exposed to five different batches (lots) of pepsin in SGF. Each lot of pepsin was of different purity (as indicated) and was adjusted to 0.32% wt/vol. Data are from Herman et al. (2005).

**Figure 2:**
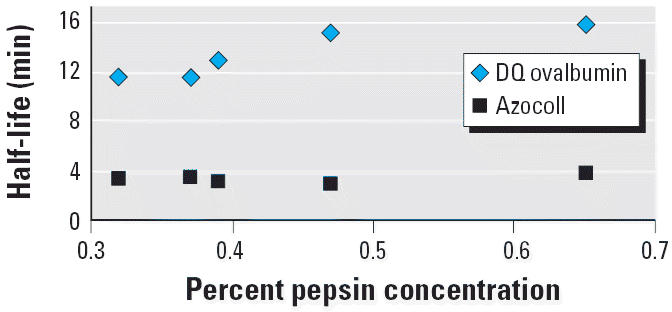
Protein stability to different pepsin concentrations: half-lives for the reporter substrates DQ ovalbumin (50 μg/mL) and azocoll (500 μg/mL) when exposed to five different concentrations of pepsin. Data are from Herman et al. (2005).

**Figure 3:**
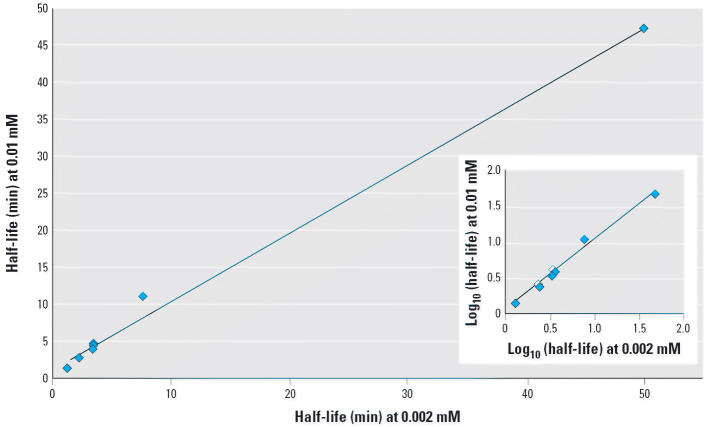
Protein stability at different substrate concentrations: relationship between half-lives of lysozyme, ovalbumin, Ara h 2, concanavalin A, concanavalin A beta subunit, concanavalin A digestion fragment, and Ara h 2 digestion fragment (in ascending order of half-lives) when exposed to SGF at 5-fold different concentrations; *y* = 0.925*x* + 1.0663, *R*^2^ = 0.9936. Inset illustrates data on a logarithmic scale for better discrimination of individual data points; *y* = 0.09704*x* + 0.0726, *R*^2^ = 0.987. Data are from Herman et al. (2004).

In addition to variable interpretations of SGF data, researchers have also deviated from SGF specifications (U.S. Pharmacopeia 2000) when conducting pepsin digestion assays, including alteration of the pepsin concentration and the pH (e.g., Bannon et al. 2002; Dearman et al. 2002; Thomas et al. 2004). Also, researchers have varied the initial concentration of substrate protein that is included in the SGF assay (e.g., Bannon et al. 2002; Fu et al. 2002; Reed et al. 1996; Thomas et al. 2004). To investigate variations in pepsin preparation (different lots with differing purity and specific activity), pepsin concentration, and substrate protein concentration, we conducted a study in our laboratory with two different protein substrates and five different pepsin lots (Herman et al. 2005). Results indicated that variation among pepsin lots, and significant variation in pepsin concentration (0.32–0.65%) and substrate concentration (in the low micromolar range), did not substantially affect estimated half-lives, although low purity pepsin lots (< 80%) had moderately lower catalytic power (Figures 1 and 2). Likewise, similar half-life estimates were obtained in a study where the initial protein substrate concentration was varied 5-fold (Herman et al. 2004; Figure 3). This is not an unexpected result when one of the reagents (enzyme) is in substantial excess of the other reagent (protein substrate) (Alexander and Scow 1989; Duggleby 2001; Boesten et al. 2005; Rawn 1989). In addition, results from alternative protein-quantification methods (chromophore or fluorophore release from reporter substrates) agreed with half-life estimates derived from SDS–PAGE and densitometry, including independent data obtained from the literature (Herman et al. 2005; Takagi et al. 2003). Baderschneider et al. (2002) also validated the SDS–PAGE and densitometry analysis using an alternate analytical method (HPLC). Together these studies indicate that a kinetic approach to characterizing stability in SGF is robust to typical variations that might occur in the digestion procedure and, unlike the time-to-disappearance approach, independent of the method used to track protein decay.

## Assay Relevance

A final requirement for a valid assay is relevance to the property that is of interest. Three aspects related to the relevance of SGF assay results are discussed here: accuracy of tracking *in vitro* stability in SGF, relevance to *in vivo* stability, and correlation with allergenicity.

The first level of relevance is the ability of the assay to reflect the stability of a substrate protein in the SGF assay. The alignment of the kinetic interpretation of data with established enzyme and biodegradation literature (see references in “Kinetic Data Analysis”), in combination with cross-validation studies on the analytical procedures (Baderschneider et al. 2002; Herman et al. 2005), indicate that this approach is relevant to protein stability in SGF.

A second aspect of relevance is how well the SGF assay reflects stability *in vivo*. It is widely acknowledged that the SGF assay may not adequately simulate *in vivo* gastric digestion, in part because *in vivo* digestion is inherently variable across individuals and within individuals over time (e.g., Bannon et al. 2002; Burnett et al. 2002; Chikwamba et al. 2003; Mendelsohn et al. 2003). Although mimicry is not required for this assay, it should, at a minimum, index a relevant process, in this case *in vivo* stability of the protein before presentation to the immune system. However, data exist indicating that highly SGF-digestible proteins can induce immune responses (Dearman et al. 2002; Kimber and Dearman 2002) and survive *in vivo* digestion intact or as digestion fragments (Chowdhury et al. 2003, Lutz et al. 2005), but data are not yet extensive enough to reach a final conclusion on the relevance of the SGF assay to *in vivo* stability.

A third and most important level of relevance is how well SGF assay results correlate with allergenicity. It is generally acknowledged that SGF stability results are an imperfect predictor of allergenic potential (e.g., Bannon et al. 2002, 2003; Fu et al. 2002; Goodman et al. 2005; Metcalfe 2005; Thomas et al. 2004). This is true regardless of whether one considers the most stable digestion fragment and protein fraction or only the parent protein substrate. Although some studies support a correlation between stability in SGF and allergenicity (e.g., Astwood et al. 1996; Koppelman et al. 2005; van Ree 2002), other studies show a poor relationship (e.g., Diaz-Perales et al. 2003; Fu et al. 2002; Herman et al. 2004; Lee et al. 2005; Metcalfe 2005; Murtagh et al. 2002; Vieths et al. 1999; Yagami et al. 2000). This inconsistency has been largely attributed to a lack of standardized methods (e.g., Bannon et al. 2002, 2003; Metcalfe 2005; Thomas et al. 2004), but it is clear that other factors also contribute to the disparate findings.

Although purified samples of transgenic proteins are tested for biochemical and biological equivalence to plant-produced proteins (Fuchs et al. 1993; Gao et al. 2004; Gustafson et al. 1997), reference allergens and non-allergens typically have not been subjected to this level of rigor. This may result in undetected structural changes to reference proteins during purification that alter their susceptibility to proteases [e.g., chemically reduced state of peanut allergen Ara h 2 (Sen et al. 2002; Thomas et al. 2004) and heat denaturation (Takagi et al. 2003)]. In addition, the array of proteins and protein types that are chosen for inclusion in a validation study can bias interpretation (Fu et al. 2002). Clearly, there are many examples of pepsin-unstable allergens and pepsin-stable nonallergens (e.g., Diaz-Perales et al. 2003; Fu et al. 2002; Herman et al. 2004; Lee et al. 2005; Murtagh et al. 2002; Vieths et al. 1999; Yagami et al. 2000). One potential explanation for the allergenicity of pepsin-unstable proteins is possible absorption in the mouth. Absorption by the buccal mucosa would bypass exposure to gastric fluid (Dirks et al. 2005; Poulsen 2005). A second possible explanation for survival of pepsin-labile proteins is that components of the food matrix shield certain proteins from the gastric environment (Chikwamba et al. 2003).

Multiple modifications of the standard SGF recipe (Astwood et al. 1996; U.S. Pharmacopeia 2000) have been proposed and used to evaluate allergenicity potential (e.g., Reed et al. 1996; Takagi et al. 2003; Thomas et al. 2004); however, no improvement in the predictive power of these modified assays has been reported. If modifications to the SGF recipe are to be adopted, we suggest that they not only should be theoretically appealing but also should be accompanied by empirical data supporting the improved relevance of the modified assay to allergenicity assessment.

## Summary

Although there is growing evidence that the SGF assay design and analysis can be standardized so that results are both reproducible and robust, the relevance of the assay to both *in vivo* digestion and allergenic potential remains uncertain. It is generally accepted that SGF stability should be considered in the weight-of-evidence assessment of allergenic potential, but it should be weighted lower than the source of the gene and structural similarity with known allergens (Lewis et al. 2005). Although some data are now available using kinetic analyses (Baderschneider et al. 2002; Herman et al. 2003, 2004, 2005; Takagi et al. 2003), additional kinetic results for well-characterized preparations of known allergens and nonallergens will need to be evaluated in the SGF assay using appropriate analytical methods and interpretation before the true predictive value of this assay is understood.
